# Evaluation of the larvicidal potential of root and leaf extracts of *Saussurea costus* (Falc.) Lipsch*.* against three mosquito vectors:*Anopheles stephensi*, *Aedes aegypti,* and *Culex quinquefasciatus*

**DOI:** 10.1590/0037-8682-0018-2019

**Published:** 2020-03-16

**Authors:** Sofi Imtiyaz Ali, Venugopalan Venkatesalu

**Affiliations:** 1 Department of Botany, Annamalai University, Annamalainagar - 608 002, Tamil Nadu, India.

**Keywords:** Anopheles stephensi, Aedes aegypti, Culex quinquefasciatus, Saussurea costus, Vectors, Larvicidal activity

## Abstract

**INTRODUCTION:**

The larvicidal potential of *Saussurea costus* (Falc.) Lipsch. was studied against the early 4^th^ instar larvae of *Anopheles stephensi* Liston., *Aedes aegypti* Linn.,and *Culex quinquefasciatus* Say. because of the emergence of mosquito resistance to conventional synthetic insecticides.

**METHODS:**

At concentrations of 12.5-200 ppm, larvicidal activities were studied under laboratory conditions.

**RESULTS:**

After 24 h of exposure, the methanol extract of the roots recorded the highest larvicidal activity against *An. stephensi,* with LC_50_ and LC_90_values of 7.96 and 34.39 ppm, respectively.

**CONCLUSIONS:**

We are developing potent larvicidal compound(s) from *S. costus* for controlling the mosquito larval population.

The Culicidae familyis comprised of approximately 3500 mosquito species. The genera *Anopheles, Aedes,* and *Culex* act as vectors of various diseases, such as encephalitis, chikungunya, dengue fever, filariasis, and malaria^1^, which compromise human health^2^.The important vector of malaria in the urban districts of India and other West Asian countries is *Anopheles stephensi* ^3^, which afflicts 36% of people situated in tropical and subtropical regions^4^. The female mosquitoes of the genus *Aedes* transmit the viruses of dengue, zika, and chikungunya fever in the tropical and subtropical urban regions of the world. At present, approximately 2500 million people are facing the threat of dengue fever and nearly 50 million cases are recorded every year^5^. The parasitic filarial nematodes (roundworms - Family Filarioidea) *Wuchereria bancrofti* (90% of infections), *Brugia malayi* (9% of infections), and *Brugia timori* (1% infections) cause lymphatic filariasis, for which the vector is *Culex quinquefasciatus*. There are approximately 120 million prevalent infections that are caused by these filarial worms, most of which are due to *W. bancrofti* ^6^. 

Mosquito control is facing timely challenges due to the inadequate success of bio-control programs and emergence of resistance to the conventional synthetic insecticides, which have necessitated the need to investigate and develop unconventional strategies by means of eco-friendly, environmentally safe, and biodegradable products as mosquito larvicides^7^. Natural products from plants have been evaluated as prototypes for new insecticidal agents, as they comprise a rich source of bioactive compounds that are potentially suitable for utilization in integrated management programs^8^. Consequently, the present study was undertaken to investigate the larvicidal potential of root and leaf extracts of *S. costus* against the early 4^th^instar larvae of *An. stephensi*, *Ae. aegypti,* and *Cx. quinquefasciatus,* as possible control measures to prevent the incidence of vector-borne diseases. This is the first study of its kind, reporting the larvicidal activities of root and leaf extracts of *S. costs* against the tested mosquito vectors. 

The roots and leaves of *Saussurea costus* (Falc.) Lipsch*.* were gathered in the month of August, 2016, from Jahama (34.198°N 74.364°E), the Baramulla district, Jammu and Kashmir, India. Then, 500 g of powdered plant material that was packed inside a Soxhlet apparatus was subjected to 72 h of successive extraction using threefold of solvent systems, like petroleum ether, chloroform, ethyl acetate, and methanol. The pooled extracts were evaporated under reduced pressure at 40 ^o^C by a rotary evaporator (Heidolf-Germany) and stored at 4 ^o^C until further assay. The voucher specimen (AUBOT#347) is deposited at the herbarium, Department of Botany, Annamalai University.

The eggs of *Anopheles stephensi* Liston. and *Aedes aegypti* Linn., and the egg rafts of *Culex quinquefasciatus* Say. were procured from the Center for Research in Medical Entomology (ICMR-Government of India), Madurai, and reared in the laboratory (29±3 ^o^C, 75 to 85% RH) by feeding with Brewer’s yeast/dog biscuits (1:3). The eggs/egg rafts were used for a larvicidal bioassay at the early 4^th^ instar larval stage, as per the standard procedures recommended by the WHO^9^. The mortality of the larvae was also checked using control groups (water and DMSO). Probit analyses (SPSS, version 21.0) were used for calculating the lethal concentrations, LC_50_ and LC_90_, and their 95% confidence limit of upper and lower confidence levels.

To determine whether *S. costus* possess a larvicidal effect against the early 4^th^ instar larvae of the selected mosquito species, the larvae were exposed to different root and leaf extracts of *S. costus* in a concentration dependent manner during 12 and 24 h of exposure. Varied levels of larvicidal activities were observed for all tested extracts, while there were no recorded larval mortalities during the control treatments (DMSO and water). Among the different extracts of *S. costus* roots*,* the methanol extract recorded the highest larval mortality. After 12 h of exposure, the root methanol extract had LC_50_ and LC­_90_ values of 10.70 and 53.91 ppm for *An. stephensi*,14.97 and 108.15 ppm for *Ae. aegypti,* and 23.90 and 185.03 ppm for *Cx. quinquefasciatus*, respectively. After 24 h of exposure, the root methanol extract had LC_50_ and LC­_90_ values of 7.96 and34.39 ppm for *An. stephensi,*10.79 and 60.71 ppm for *Ae. aegypti,* and 15.31 and 105.63 ppm for *Cx. quinquefasciatus*, respectively. The larvicidal activity of the methanol extract was followed by that of petroleum ether >chloroform and ethyl acetate extracts of *S. costus* after 24 h of exposure (Figure 1).

**FIGURE 1:**
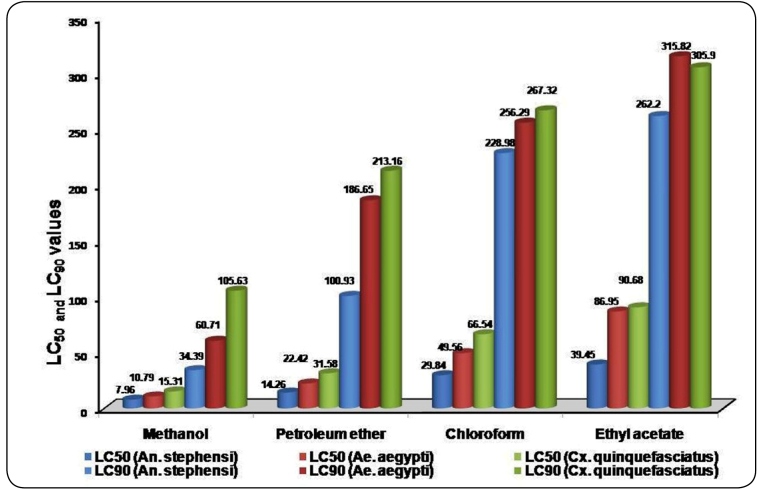
Larvicidal potential of *Saussurea costus* root extracts against *An. stephensi, Ae. aegypti,* and *Cx. quinquefasciatus.*

The larvicidal activity of the petroleum ether leaf extract of *S. costus* was higher than that of the other leaf extracts tested against *An. stephensi, Ae. Aegypti,* and *Cx. quinquefasciatus.* After 12 h of exposure, the petroleum ether leaf extract had LC_50_ and LC_­90_ values of 27.83 and184.82 ppm for *An. stephensi*,62.73 and 249.03 ppm for *Ae. aegypti,* and 87.56 and 269.59 ppm for *Cx. quinquefasciatus*, respectively. After 24 h of exposure, the petroleum ether leaf extract had LC_50_ and LC­_90_ values of 17.72 and 138.32 ppm for *An. stephensi*, 23.49 and 172.91 ppm for *Ae. aegypti,* and 50.12 and 165.77 ppm for *Cx. quinquefasciatus*, respectively. The larvicidal activity of the petroleum ether extract was followed by that of methanol >chloroform and ethyl acetate extracts after 24 h of exposure (Figure 2).

**FIGURE 2:**
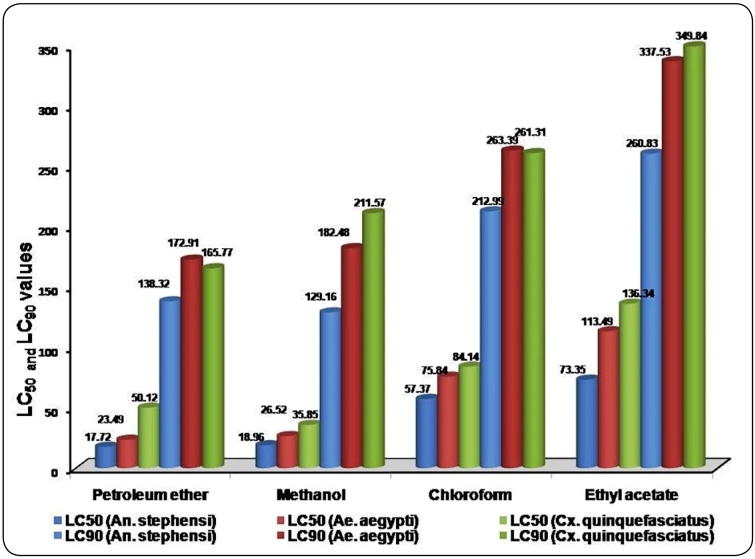
Larvicidal potential of *Saussurea costus* leaf extracts against *An. stephensi, Ae. aegypti,* and *Cx. quinquefasciatus.*

Dehydrocostus lactone and costunolide, isolated from the essential oils of the roots of *S. costus,* strongly support the theory that *S. costus* could be an effective larvicidal plant, as they exhibit strong larvicidal activity against *Ae. albopictus* with LC_50_ values of 2.34 and 3.26 μg/mL, respectively^10^. In addition, *An. stephensi* and *Ae. aegypti* larvae were found to be more susceptible to plant extracts than other mosquito species, since the methanol extract of *Terminalia chebula* was more effective against *An. stephensi* (LC_50_ = 87.13 ppm) and *Ae. Aegypti* (LC_50_ = 93.24 ppm) than *Cx. Quinquefasciatus* (LC_50_ = 111.98 ppm)^11^. Additionally, Ramya et al^12^observed the highest larval mortality from the ethyl acetate fraction of a leaf extract of *Catharanthus roseus*, followed by the methanol fraction against the I, II, III, IV, V, and VI instar larvae of *Helicoverpa armigera*. Similar to our results, among the different extracts tested, the methanol extract of the roots of *R. cordifolia* was more potent against *Cx. quinquefasciatus,* with LC_50_ and LC_90_ values of 95.69 and 347.96 mg/L, respectively^13^. Moreover, the methanol extract of *Andrographis echioides* had a higher toxicity against *Ae. Aegypti* (LC_50_ = 93.00 and LC_90_ = 83.06 ppm) than against *Cx. quinquefasciatus* (LC_90_ = 171.81 and LC_90_ = 171.76 ppm)^14^.

Likewise, the petroleum ether extract of the leaves of *Ruta graveolens* showed the highest larvicidal activity against *An. stephensi,* having an LC_50_ value of 31.89 µg/mL and LC_90_ value of 66.96 µg/mL, while it had an LC_50_ value of 66.96 µg/mL against *Ae. aegypti* after 24 h of exposure^15^ *.* In another study, the larvicidal efficacy of the ethanol, acetone, and petroleum ether extracts of the leaves of *Tribulus terrestris* were studied against 3^rd^ instar larvae of *Ae. aegypti*. Among the other tested extracts, the petroleum ether extract was found to be the most effective, with an LC_50_ value of 64.6 ppm^16^.

The present work demonstrates that *S. costus* could be considered as a novel and effective source for use in vector control programs because of its biocidic effect against the larval stages of *An. stephensi*, *Ae. aegypti,* and *Cx. quinquefasciatus* at low concentrations. The compound(s) responsible for the larvicidal activity should be isolated from the methanol extract of the roots of *S. costus* through bioassay-guided fractionation, which is under way in our laboratory. 
